# Beyond DNA editing: how Cas13 redefined programmable RNA manipulation and what still limits its therapeutic promise

**DOI:** 10.1093/nar/gkag616

**Published:** 2026-06-23

**Authors:** Saiying Hou, Wanda Bi, Wenyi Liu, Henghai Nie, Jianxin Jiang, Ling Zeng

**Affiliations:** State Key Laboratory of Trauma and Chemical Poisoning, Department of Trauma Medical Center, Daping Hospital, Army Medical University, Changjiang Branch Road 10, Daping Street, Yuzhong District, Chongqing 400042, China; State Key Laboratory of Trauma and Chemical Poisoning, Department of Trauma Medical Center, Daping Hospital, Army Medical University, Changjiang Branch Road 10, Daping Street, Yuzhong District, Chongqing 400042, China; State Key Laboratory of Trauma and Chemical Poisoning, Department of Trauma Medical Center, Daping Hospital, Army Medical University, Changjiang Branch Road 10, Daping Street, Yuzhong District, Chongqing 400042, China; State Key Laboratory of Trauma and Chemical Poisoning, Department of Trauma Medical Center, Daping Hospital, Army Medical University, Changjiang Branch Road 10, Daping Street, Yuzhong District, Chongqing 400042, China; State Key Laboratory of Trauma and Chemical Poisoning, Department of Trauma Medical Center, Daping Hospital, Army Medical University, Changjiang Branch Road 10, Daping Street, Yuzhong District, Chongqing 400042, China; State Key Laboratory of Trauma and Chemical Poisoning, Department of Trauma Medical Center, Daping Hospital, Army Medical University, Changjiang Branch Road 10, Daping Street, Yuzhong District, Chongqing 400042, China

## Abstract

The Type VI Clustered Regularly Interspaced Short Palindromic Repeats (CRISPR)-Cas13 system, evolved from prokaryotic immunity, has become a versatile, programmable RNA-targeting platform with broad biotechnological potential. Guided by CRISPR RNA (crRNA), Cas13 cleaves single-stranded RNA via higher eukaryotic and prokaryotic nucleotide-binding domains, exerting specific (*cis*) and nonspecific (*trans*) collateral cleavage, which enables ultrasensitive nucleic acid detection while introducing cytotoxicity risks in eukaryotic cells. Diversification of Cas13 subtypes, including compact variants, enhances targetability and delivery compatibility, and inhibitory strategies (anti-CRISPR proteins, crRNA mimicry/degradation) enable activity modulation for improved safety. Building on mechanistic foundations, Cas13 is repurposed for targeted RNA knockdown, nucleic acid diagnostics, live-cell RNA imaging with catalytically inactive variants, programmable RNA base editing through deaminase fusions, splicing regulation, epitranscriptomic editing of multiple RNA chemical marks, interaction mapping of RNA–protein and RNA–RNA networks, and translational control, with preliminary clinical translation in antiviral therapies, pathogenic transcript correction, and cancer therapy. Furthermore, Cas13-integrated diagnostics and functional genomics are accelerating biomarker discovery and personalized treatment. Nevertheless, successful clinical translation hinges on overcoming critical bottlenecks, including tissue-specific delivery, mitigation of collateral cytotoxicity, and management of host immunogenicity. This review synthesizes Cas13 classification, structure–function principles, regulatory inhibitors, application modalities, and translational challenges to inform next-generation engineering and responsible deployment of RNA-targeted technologies.

## Graphical Abstract

**Graphical Abstract:**
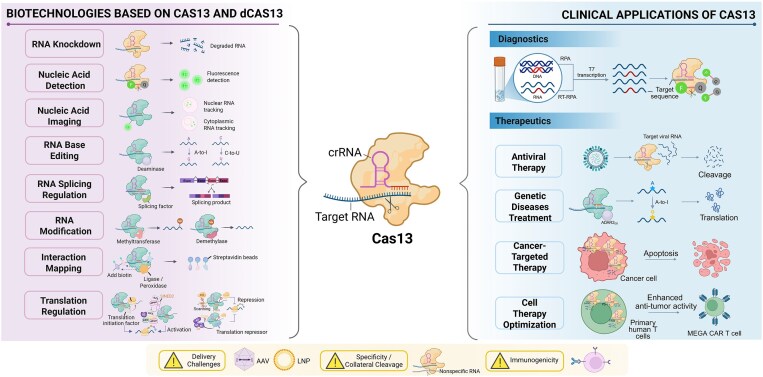

## Introduction

**Figure 1.**
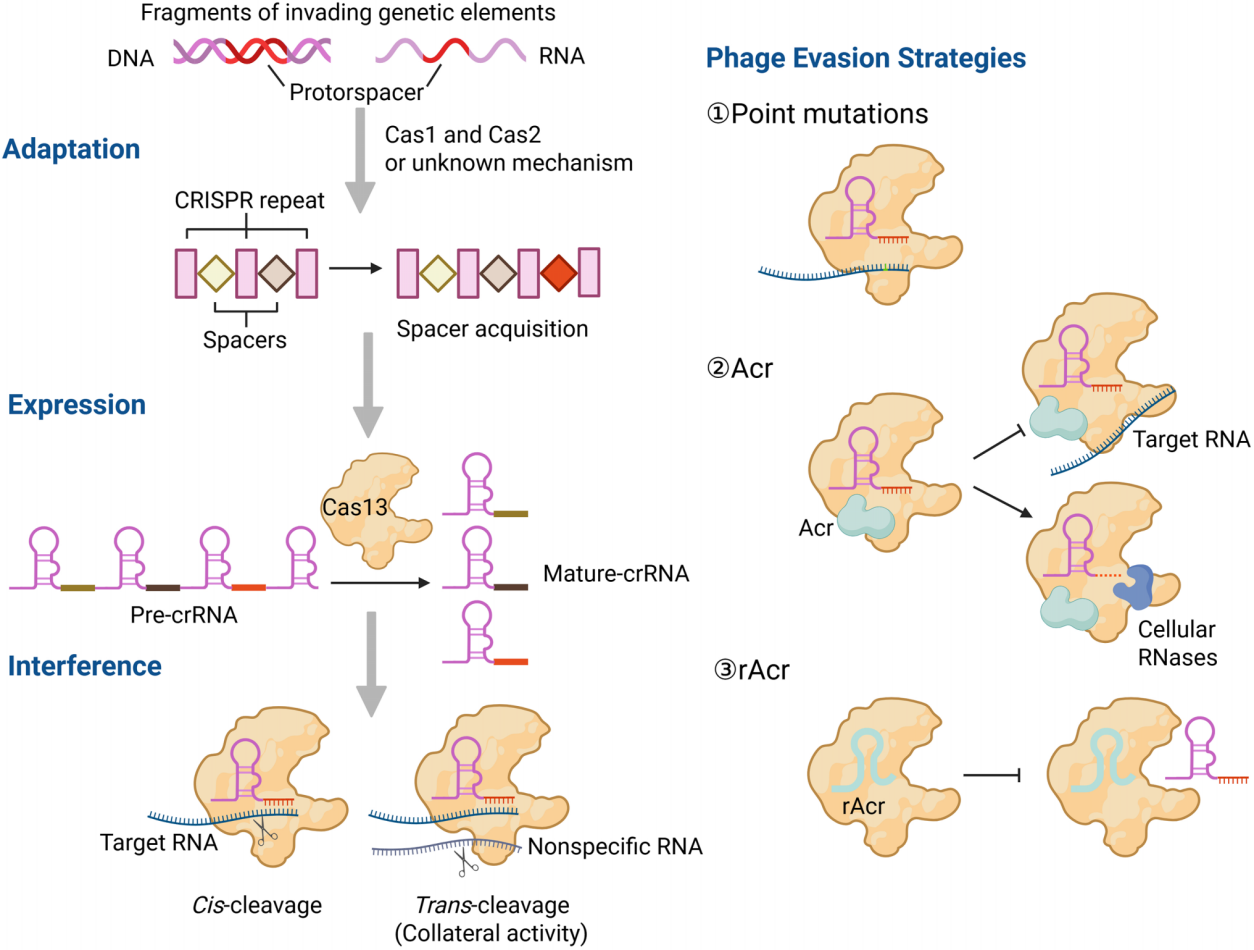
Adaptive immunity of the CRISPR-Cas13 system. Adaptation: fragments from invading genetic elements are processed, with the protospacer segment excised and integrated as a new spacer into the CRISPR array. This process is mediated by Cas1 and Cas2 or through other uncharacterized mechanisms. Rectangles represent CRISPR repeats, while diamonds denote acquired spacers. Expression: the CRISPR array is transcribed into pre-crRNA, which is subsequently processed by Cas13 into mature crRNAs. Each mature crRNA comprises a single spacer sequence flanked by a direct repeat (DR). Interference: the mature crRNA guides Cas13 to recognize a complementary target RNA, triggering sequence-specific *cis*-cleavage of the target and subsequent non-specific *trans*-cleavage (collateral activity) of surrounding RNAs. Phage-encoded mechanisms to evade Cas13 immunity are categorized into three core strategies: (i) protospacer mutation, (ii) anti-CRISPR proteins, and (iii) RNA-based anti-CRISPRs. Created in BioRender. Huang, S. (2026) https://BioRender.com/urpibdf

Most bacteria and archaea possess an adaptive immune system termed the Clustered Regularly Interspaced Short Palindromic Repeats (CRISPR) and CRISPR-associated (Cas) systems, which defends against mobile genetic elements by storing sequences from past invaders and using RNA-guided nucleases to degrade matching nucleic acids [1, 2]. A canonical CRISPR locus contains Cas genes together with a CRISPR array composed of repeat-spacer units, and immunity proceeds through three connected stages: spacer acquisition from invading nucleic acids, expression and processing of precursor CRISPR RNAs (pre-crRNAs), and interference against complementary targets [3–7] (Fig. 1). In adaptation, the Cas1–Cas2 integrase complex captures protospacers from invading nucleic acids, a process sometimes assisted by accessory factors such as reverse transcriptase, Cas4, or Csn2. The excised fragment is directionally integrated as a new spacer into the CRISPR array, establishing immunological memory [8]. During expression, the array is transcribed into pre-crRNA, which is processed by Cas proteins or host RNases into mature crRNAs, each comprising a spacer and partial repeat sequence [9, 10]. In the interference stage, crRNA guides Cas effector complexes to complementary foreign nucleic acids, triggering their sequence-specific cleavage or degradation [11–13].

Structurally and functionally, CRISPR-Cas systems are categorized into Class 1 (types I, III, and IV) and Class 2 (types II, V, and VI), which comprise multiple Cas subunits and a single multidomain Cas protein, respectively [14–16]. Within Class 2, Cas9 (type II) and Cas12 (type V) function as RNA-guided DNA nucleases, whereas Cas13 (type VI) is the best-characterized RNA-directed effector. Cas13 utilizes Higher Eukaryotic and Prokaryotic Nucleotide-binding (HEPN) endoribonuclease domains, characterized by two conserved RxxxxH motifs, to catalyze single-stranded RNA (ssRNA) cleavage via arginine (R) and histidine (H) residues that facilitate phosphodiester bond hydrolysis [14, 17–20]. Unlike DNA-targeting systems that require a canonical protospacer adjacent motif (PAM), several Cas13 orthologs exhibit subtype-specific preferences for a short motif flanking the guide–target duplex, termed the protospacer flanking sequence (PFS) [20–23]. Notably, certain variants, such as Cas13d, are effectively PFS-independent, a characteristic that substantially expands the range of targetable RNA transcripts [22]. The intrinsic pre-crRNA processing activity of Cas13 is a prominent advantage and a key feature enabling its multiplex targeting capability [24]. Unlike Cas9, Cas13 can independently complete pre-crRNA maturation and processing without accessory proteins, thereby enabling simultaneous loading of multiple crRNAs to achieve specific recognition and cleavage of multiple RNA targets [25]. This provides a robust molecular foundation for the development of Cas13-based biotechnologies, including multiplex nucleic acid detection and coordinated gene expression regulation [26, 27]. However, this feature is subtype-dependent and is absent or not yet demonstrated in some compact Cas13 families, such as Cas13bt and Cas13h1 [28, 29].

These properties establish Cas13 as a versatile, programmable platform for reversible, transcript-level perturbation. The system is capable of silencing endogenous RNAs, processing multiplexed guide arrays, and amplifying diagnostic signals through collateral cleavage, while also serving as a versatile scaffold for RNA editing, imaging, splicing control, and translational modulation [30–32]. Nevertheless, the same attributes that empower Cas13 also constrain its clinical translation. Specifically, guide efficacy remains highly subtype- and context-dependent, and while collateral activity provides significant diagnostic utility, it complicates therapeutic precision. Furthermore, challenges in *in vivo* delivery and potential host immunogenicity continue to impede clinical deployment. Against this backdrop, this review synthesizes current knowledge of Cas13 classification, mechanisms, guide design, regulatory inhibition, and application classes, while critically addressing the translational bottlenecks that currently define the field.

## Overview of CRISPR-Cas13 system

### Classification of CRISPR-Cas13 systems

**Figure 2.**
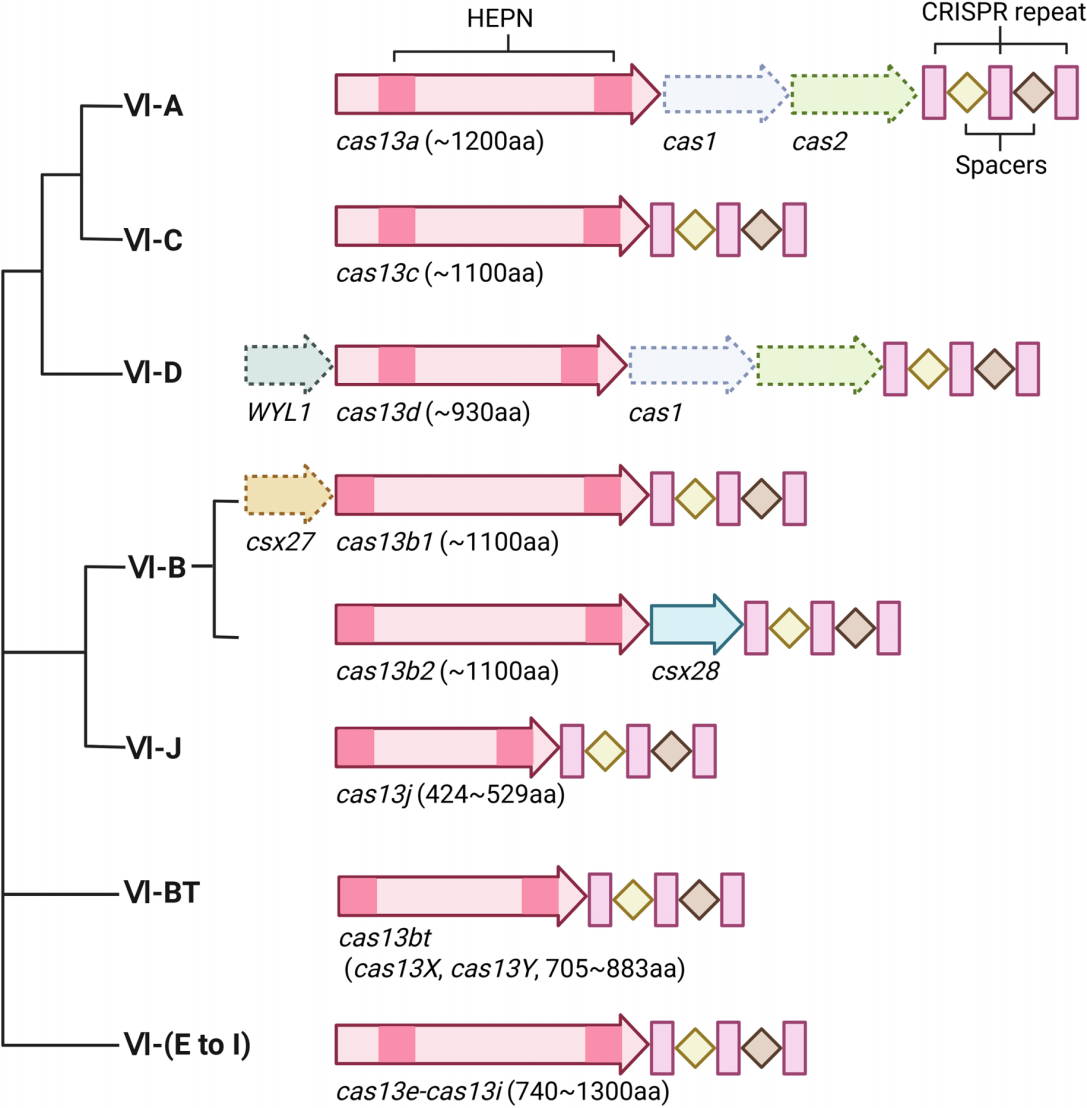
Classification and genomic architecture of Type VI CRISPR-Cas13 systems. The phylogenetic tree (left) illustrates the evolutionary relationships among various Cas13 subtypes. The corresponding genomic loci (right) depict the organization of each subtype, typically comprising a Cas effector protein with two HEPN domains and a CRISPR array. Within these effectors, shaded regions indicate the approximate positions of the conserved HEPN domains. Genes encoding Cas1, Cas2, Csx27, and WYL domain-containing proteins are present only in specific subtypes and are thus indicated with dashed outlines. Notably, Csx27 and Csx28 are proposed accessory proteins for Cas13b1 and Cas13b2, respectively. The average protein size (in amino acids, aa) is labeled beneath each representative gene. Created in BioRender. Huang, S. (2026) https://BioRender.com/urpibdf

Following the foundational categorization of Type VI systems by Shmakov *et al*., the Cas13 family has expanded significantly beyond the original Cas13a–c groups to include Cas13d, Cas13bt (Cas13X/Y), and the more recently identified Cas13e–j orthologs [14, 33–37] (Fig. 2). Across this diversity, Cas13 proteins are crRNA-guided ribonucleases characterized by two distinct catalytic operations handled by independent active sites: target-triggered RNA cleavage mediated by the conserved HEPN domains, and, in many subtypes, pre-crRNA processing driven by a separate catalytic center [3, 37]. This mechanistic distinction is highlighted by the fact that catalytically dead Cas13 (dCas13) variants, despite possessing inactivated HEPN motifs that abolish target cleavage, fully retain their capability for pre-crRNA maturation [22, 23, 38]. Unlike Cas9, which cleaves only double-stranded DNA complementary to the spacer, the activated Cas13–crRNA cleaves the complementary target RNA *in cis* and can subsequently degrade nearby non-target RNAs *in trans* through collateral (bystander) activity [39].

The first characterized Cas13a, derived from *Leptotrichia shahii* (LshCas13a), demonstrated programmable RNA-guided interference against the MS2 RNA phage in *Escherichia coli* [21]. The LshCas13a locus encodes a CRISPR array and the single effector Cas13a, which utilizes two RNA-cleaving HEPN domains, alongside Cas1/Cas2 proteins dedicated to spacer acquisition [21]. In 2017, Smargon *et al*. computationally identified Cas13b subtypes, which typically lack the Cas1/Cas2 adaptation module and instead encode accessory proteins, such as Csx27 or Csx28 [23]. Expanding the family further via CRISPR-seed-based searches, Shmakov *et al*. identified Cas13c, observing that these subtypes share minimal sequence identity beyond their conserved yet differentially positioned HEPN motifs [14]. The subsequent discovery and engineering of RfxCas13d (CasRx), from *Ruminococcus flavefaciens* XPD3002, marked a significant advancement; it demonstrated robust activity in human cells and enabled adeno-associated virus (AAV)-mediated delivery due to its compact architecture [22]. In 2021, the identification of two additional compact families, Cas13X and Cas13Y, further expanded the toolkit with systems that efficiently mediate mammalian RNA interference despite lacking accessory genes [28, 31, 32]. By mining diverse metagenomic datasets in 2022, Hu *et al*. identified five additional clades, Cas13e through Cas13i [36]. Most of these subtypes lack conserved adaptation genes but include ultracompact members, such as Cas13e3, which exhibit high interference efficiency alongside minimal collateral activity. Most recently, the Cas13j family, including LepCas13j and ChiCas13j discovered in soil metagenomes, represents the smallest Cas13 proteins reported to date [40].

### Mechanistic principles that define Cas13 performance

**Figure 3.**
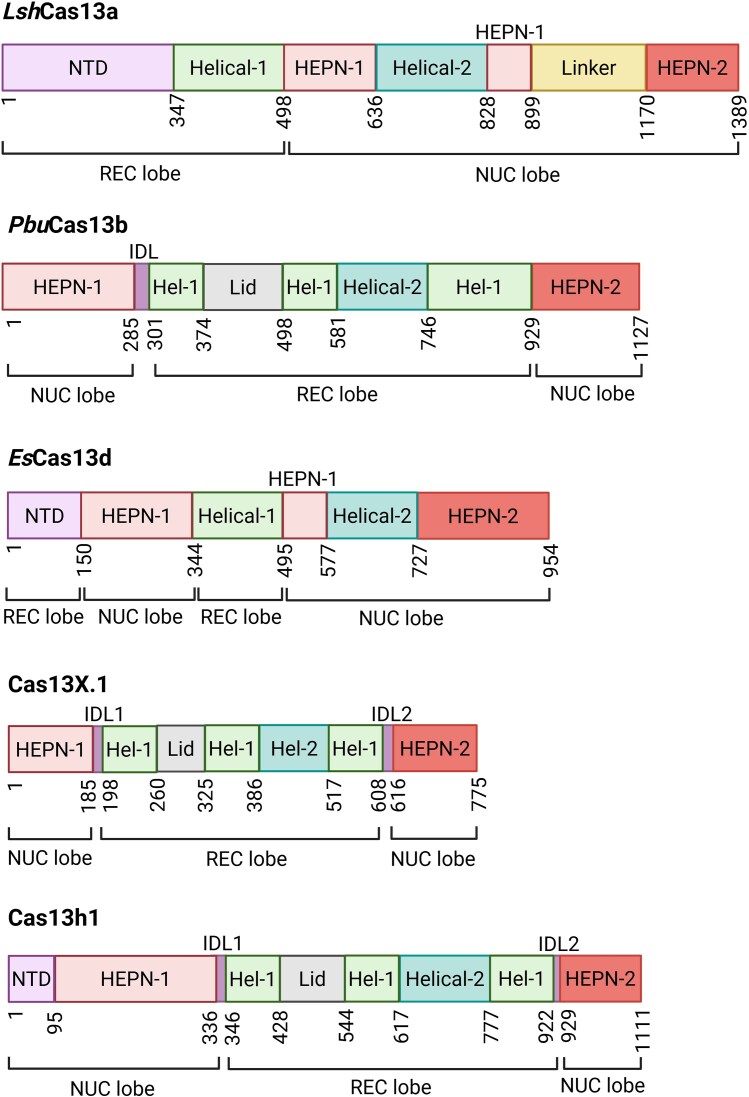
Domain organization of representative Type VI CRISPR-Cas effectors. Despite their diversity, Cas13 orthologs share a conserved bilobed architecture partitioned into REC and NUC lobes. This figure illustrates the linear domain arrangement of various Cas13 proteins, highlighting the specific domain composition of the REC and NUC lobes with corresponding amino acid positions indicated. REC, recognition lobe; NUC, nuclease lobe; NTD, N–terminal domain; IDL, inter-domain linker. Created in BioRender. Huang, S. (2026) https://BioRender.com/urpibdf

Although Cas13 orthologs exhibit limited primary sequence similarity, they share a conserved bilobed architecture characteristic of single-effector Cas proteins. This structure is partitioned into a recognition lobe (REC, comprising an N-terminal domain and a Helical-1 domain) that mediates crRNA binding, and a nuclease lobe (NUC, composed of Helical-2 and split HEPN-1/HEPN-2 domains) that facilitates target RNA accommodation and catalytic cleavage [3, 39]. This conserved architecture underscores how such divergent enzymes maintain programmable RNA-targeting capabilities, yet accommodates significant subtype-specific variations in guide architecture, target recognition, and accessory-factor requirements. Detailed structural representations of canonical members from each subtype are discussed below and visualized in Fig. 3.

Cas13a remains the most thoroughly characterized subtype mechanistically [3]. Based on target cleavage specificity, Cas13a orthologs are categorized into uracil (U)-cleaving and adenine (A)-cleaving subfamilies [21]. Structural studies of *L. shahii* Cas13a (LshCas13a) and *L. buccalis* Cas13a (LbuCas13a) have established that the DR forms a defined stem-loop, where the bulge nucleotides are indispensable for pre-crRNA processing and target cleavage [38, 39, 41–43]. Cas13a systems also provide the clearest framework for understanding PFS and tag:anti-tag logic. In LshCas13a, cleavage activation is governed by a 3′-H (non-G) PFS [21, 44], whereas extended complementarity between the crRNA 5′ tag and the target 3′ anti-tag region induces an inhibitory conformational change in the Cas13–crRNA ribonucleoprotein (RNP) complex, thereby abrogating nuclease activity [3, 45]. Specifically, a study on LseCas13a has demonstrated that the 3′-terminal GTT/GTTT motifs flanking the protospacer serve as endogenous anti-tags; these motifs completely eliminate both *cis* and *trans* activities when complementarity with the crRNA tag reaches 7 bp [46]. By preventing autoimmunity and mediating bacterial immune regulation, this tag:anti-tag checkpoint exemplifies the mechanistic coupling of target recognition to regulatory discrimination, where Cas13a activation depends on a combination of flanking-sequence preference and the absence of inhibitory tag:anti-tag complementarity.

Cas13b exhibits critical structural and mechanistic divergences from Cas13a that significantly influence its biotechnological implementation. Structural analyses of *Bergeyella zoohelcum* Cas13b (BzCas13b) and *Prevotella buccae* Cas13b (PbuCas13b) reveal that Cas13b crRNAs adopt a 5′-spacer orientation, directly contrasting with the 3′-spacer orientation of Cas13a, and rely on extensive protein interactions outside the HEPN-2 domain [47, 48]. Additionally, BzCas13b interference is strictly governed by dual PFS constraints (a 5′ non-C PFS and a 3′ NAN/NNA PFS) and a pronounced nucleolytic bias toward pyrimidines [23]. Beyond intrinsic RNA cleavage, Cas13b is uniquely modulated by transmembrane accessory proteins: VI-B1 loci variably encode the inhibitory Csx27, whereas VI-B2 loci consistently encode Csx28 [23, 39]. Notably, Csx28 functions as a downstream membrane-associated effector that triggers inner-membrane depolarization upon Cas13b-mediated phage sensing [49]. This integration with membrane-level defense outputs broadens the conceptual framework of type VI CRISPR systems, demonstrating that Cas13-centered immunity operates as a complex, multilayered physiological response rather than an isolated nuclease.

Cas13d is the most consequential subtype for mammalian engineering due to its compactness, robust activity, and broad targeting flexibility [25, 50–52]. Structures of *Eubacterium siraeum* Cas13d (EsCas13d) and *Ruminococcus* sp. Cas13d (Ur/RspCas13d) support the standard REC/NUC framework but reveal distinctive features, including a hinge-like HEPN-1 scaffold and subtype-specific guide interactions [25, 50]. Importantly, Cas13d target cleavage is activated by spacer-complementary RNA, and Mg^2+^ can enhance cleavage efficiency by stabilizing the active RNP conformation rather than serving as a direct catalytic cofactor for HEPN-mediated RNA cleavage [22, 25, 52]. Cas13d preferentially cleaves uracil-rich ssRNA with low secondary structure and is generally free of PFS restrictions, providing a significant advantage for flexible RNA targeting [52]. Most VI-D loci also encode a neighboring WYL-domain accessory protein with additional DNA-binding motifs, which enhances Cas13d-mediated cleavage in a dose-dependent manner [51]. Together, these features help explain why Cas13d has become the dominant scaffold for *in vivo* knockdown and dCas13-based editing applications.

As an ultracompact type VI effector with substantial *in vivo* delivery potential, Cas13bt (Cas13X/Cas13Y) maintains a canonical bilobed architecture partitioned into REC and NUC lobes, yet Cas13bt3’s (Cas13X.1) 775-amino-acid footprint is significantly smaller than canonical Cas13b orthologs due to the absence of large domain insertions [28]. Mechanistically, Cas13bt interference is restricted by a 5′-D (non-C) PFS and notably lacks intrinsic pre-crRNA processing activity *in vitro*, implying a reliance on host or auxiliary factors [28, 31]. Similarly, the recently identified Cas13h family comprises eight distinct members exhibiting robust RNA-targeting capabilities, where Cas13h1 leverages a central seed region to form a specialized nucleotide-binding pocket for 5′-PFS recognition [36]. While Cas13h1 cleavage is enhanced by a 5′-R (G/A) PFS, inhibited by extended tag:anti-tag complementarity, and similarly lacks intrinsic pre-crRNA processing, its full translational realization remains hindered by critical unresolved questions [28, 29]. Specifically, future investigations must delineate the mismatch tolerance of the Cas13h seed region, identify its *in vivo* processing mediators, resolve the structural basis of PFS-dependent modulation, and characterize the functional variance across the entire Cas13h clade.

### Challenges and optimization strategies of crRNA design

The optimization of crRNA design represents the critical rate-limiting step in translating Cas13 mechanisms into robust biotechnological tools. In practice, guide performance is dictated by a complex interplay of variables, including subtype identity, spacer geometry, mismatch discrimination, target accessibility, and intracellular microenvironment. Consequently, guide sequences exhibiting high potency within one specific subtype, host cell line, or experimental paradigm frequently fail to translate effectively to others. Therefore, Cas13 guide formulation cannot be reduced to a universal predictive algorithm; rather, it constitutes a highly bespoke, subtype- and context-specific engineering challenge.

Cas13 subtype-specific characteristics dictate crRNA design principles. Cas13a typically uses a 5′ DR handle followed by a 3′ spacer, where the DR stem-loop structure is indispensable for nuclease activation [42]. In contrast, Cas13b utilizes a 5′-spacer orientation and retains specific DR complementary regions to maintain structural stability [39]. Beyond orientation, spacer length requirements are similarly divergent: while RfxCas13d displays maximal knockdown efficiency with 23–30 nt guides [53], other variants exhibit a broader functional range (20–28 nt) with minimal impact on activity [54]. Targeting accuracy and efficiency are further determined by spacer base composition, mismatch tolerance, and crRNA secondary structure. For RfxCas13d, a central “seed” region (positions 15–21, centered at 18) exhibits heightened mismatch sensitivity, where optimal activity correlates with a 45%–55% core GC content and higher minimum free energy [53, 55]. Conversely, Cas13a recognizes a non-G PFS and features a 6–11 nt mismatch-sensitive window, with broader tolerance observed at the 3′ terminus [56]. Furthermore, the target site within the transcript significantly influences performance: CDS-targeted guides often outperform those targeting UTRs or introns, likely because RNP access is modulated by spliceosome occupancy and RNA-binding protein (RBP) interference [53].

Current optimization strategies integrate chemical engineering, predictive modeling, and application-aware design. Chemical modifications, such as 2′-O-methyl and phosphorothioate linkages, significantly enhance guide stability and specificity [57]. Furthermore, secondary-structure-informed strategies exploit the innate preference of Cas13 for accessible single-stranded regions [54]. While advanced crRNA prediction tools, such as RFcombined-based models, convolutional neural-network approaches (CNNs), and DeepCas13 have improved guide prioritization within specific subtypes, a universal, cross-subtype predictor remains elusive [53, 55, 58, 59]. This limitation is particularly critical for clinical translation, where diagnostic assays, antiviral interventions, allele-specific silencing, and transcript correction each impose distinct requirements for sensitivity, specificity, and mismatch tolerance. For instance, the ADAPT system enables high-sensitivity diagnosis for over 1900 vertebrate viruses but relies on comprehensive viral genome data, which constrain its accuracy for emerging or under-sequenced pathogens [56]. Conversely, Shembrey *et al*.’s synthetic mismatch strategy has achieved single-base precision in silencing oncogenic transcripts with minimal off-target effects on wild-type alleles [60].

Moving forward, crRNA design must be repositioned as a fundamental design axis rather than a secondary optimization step. The field currently lacks a cohesive framework that integrates subtype-specific structural logic, RNA accessibility, and cellular context with off-target risks across diverse applications. Future advancements will likely emerge from synthesizing mechanistic structural insights with large-scale functional screening across diverse orthologs and biological landscapes, rather than the mere accumulation of incremental empirical rules. For clinical translation, this necessitates subtype-aware predictive models aligned with specific therapeutic or diagnostic objectives, moving beyond monolithic benchmarks of “activity.” This divergence is most evident when contrasting diagnostics and therapeutics: while diagnostic guides prioritize maximal target-triggered activation, therapeutic modalities mandate a stringent balance between potency and collateral risk, requiring high-fidelity discrimination among closely related isoforms while navigating structurally occluded transcript regions. Ultimately, a mature Cas13 landscape demands differentiated design standards tailored to the unique functional requirements of each biological inquiry.

### Cas13 collateral activity: control rather than abolition

Collateral cleavage is the defining attribute that both empowers and complicates Cas13. Upon guide-dependent recognition of a complementary RNA, Cas13 undergoes a conformational change that not only cleaves the target but also directly degrades proximal RNAs *in trans* through non-specific cleavage [61] (Fig. 1). In its natural prokaryotic context, this activity can eliminate both phage and host transcripts, thereby arresting bacterial growth and disrupting the viral infection cycle [62]. Structurally, the HEPN catalytic centers are positioned away from the guide-target duplex, such that target binding allosterically activates these RNase domains to catalyze both sequence-specific cleavage of the target RNA (*in cis*) and non-specific degradation of surrounding transcripts (*in trans*), thereby manifesting as collateral activity [3].

While robust *in vitro* and in prokaryotic environments, the potency and prevalence of Cas13 collateral cleavage in eukaryotes remain contentious. Early studies reported minimal non-specific RNA degradation across mammalian cells, *Drosophila*, and zebrafish, suggesting that eukaryotic cellular environments or regulatory mechanisms might attenuate such activity [22, 61, 63–65]. However, emerging evidence identifies significant collateral effects, including the degradation of co-expressed reporters, endogenous transcripts, and housekeeping genes, with the magnitude of these perturbations being highly context-dependent, governed by ortholog identity, cell-type-specific factors, and delivery dynamics [66–69]. Consequently, the current consensus suggests that collateral activity is neither absent nor inevitable, but rather a multifactorial variable that necessitates mitigation through low-dose delivery, strategic subtype selection, or the co-administration of anti-CRISPR regulators.

Translational progress further demands a rigorous distinction between conventional off-targeting and collateral *trans*-cleavage. Unlike off-targeting, which arises from partial guide-substrate complementarity, collateral cleavage represents a target-triggered, sequence-independent catalytic state [24]. Crucially, many “off-target” concerns in Cas13 systems stem from successful target recognition rather than hybridization errors. This mechanistic dichotomy underscores why collateral activity is a significant asset for diagnostic signal amplification but a fundamental liability for therapeutic precision, where promiscuous cleavage can precipitate transcriptomic instability and cytotoxic phenotypes.

Ultimately, the transformative potential of Cas13 hinges upon the structural decoupling of on-target interference from *trans*-cleavage. Future advancements must transition from the simple abrogation of this activity toward its precise spatiotemporal orchestration, necessitating the development of subtype-aware predictive models and engineered variants tailored to the unique functional requirements of either molecular diagnostics or high-fidelity transcriptomic therapy.

### Anti-CRISPRs as tunable control modules for Cas13 activity

The ongoing evolutionary arms race between bacteria and phages has driven the emergence of diverse phage-encoded mechanisms to evade CRISPR-Cas13 immunity, which can be broadly grouped into three core strategies (Fig. 1). These evasion mechanisms not only shape the co-evolutionary dynamics of bacterial and phage populations, but also reveal key vulnerabilities of the Cas13 system to guide the optimization of Cas13-based RNA manipulation tools. The primary strategy involves phage genomic mutations that disrupt crRNA-dependent target recognition [70]. Single-nucleotide variants (SNVs) within the protospacer can abolish Cas13 binding and cleavage, particularly when occurring in critical functional elements governing initial target recognition, such as the seed region or the PFS.

The most sophisticated evasion strategy is mediated by phage-encoded anti-CRISPR (Acr) proteins, which are increasingly recognized not only as immune-evasion proteins, but also as precise regulators of CRISPR effector state with significant biotechnological potential [71–74]. This perspective is particularly relevant to Cas13 because target recognition activates both sequence-specific *cis*-cleavage and non-specific *trans*-cleavage, linking nuclease activation to both antiviral defense and broader cellular consequences [3, 72]. In the Cas13 context, inhibitors therefore matter not simply as products of host-phage conflict, but as candidate control elements for tuning activity, limiting exposure, and potentially mitigating collateral toxicity in engineered systems [3, 74].

The anti-Cas13 landscape is resolving into several mechanistically distinct classes, although the evidence base is not equally strong for all reported candidates. While early reports described seven AcrVIA proteins capable of inhibiting LwaCas13a [75], subsequent work identified major methodological problems in the original experimental setup and failed to reproduce key activities [76]. Structural re-evaluation further showed that AcrVIA6 likely functions as a monomeric DNA-binding protein rather than a direct Cas13a inhibitor [77]. In contrast, AcrVIA1 is supported by robust genetic, biochemical, and structural evidence: it binds the LseCas13a–crRNA complex, occludes the target-RNA access channel, and prevents the transition to an active HEPN nuclease state [78]. This mechanism is biologically critical because activated Cas13a can trigger rapid translational shutdown through transfer RNA (tRNA) cleavage and downstream RNase activation, imposing stringent kinetic requirements on any effective inhibitor [62, 79]. Structural characterization demonstrates that AcrVIA2 acts via a mechanism distinct from AcrVIA1: it functions as a homodimer that specifically engages the Helical-I domain of LshCas13a, thereby blocking the formation of functional Cas13–crRNA RNP complexes [80]. Another validated branch is AcrVIB1, which inhibits type VI-B systems through a distinct mechanism. After its initial identification by DeepAcr-assisted screening, structural and mechanistic analysis showed that AcrVIB1 traps apo-Cas13b in an open, unproductive state in which pre-crRNA remains vulnerable to endogenous RNase attack, thereby depleting functional guides before an active RNP can form [81, 82]. Together, these findings indicate that anti-Cas13 inhibition can occur at multiple checkpoints, from pre-assembly guide depletion to post-loading channel occlusion. Analogous inhibitory logic has also been documented in other CRISPR types, including complex disassembly and covalent inactivation, suggesting that additional Cas13 inhibitory chemistries may remain undiscovered [83–85].

A major recent advance is the discovery that anti-Cas13 regulation is not confined to proteins. rAcrVIA1 is a small RNA that adopts a crRNA-like three-dimensional structure to competitively occupy the crRNA-binding site of LseCas13a [35]. Within the same locus, AcrVIA2 provides a complementary protein-based layer of inhibition, defining a compact, multi-component anti-Cas13 module [35]. These findings reinforce the importance of structural mimicry as a recurring theme in CRISPR antagonism and highlight a discovery challenge: functionally relevant inhibitors may evade sequence-based searches if they rely solely on RNA structural features [35–87].

Looking forward, the translational value of anti-Cas13 systems lies in their potential to provide temporal control, reversible shutdown, and safer deployment of RNA-targeting technologies [3, 63, 74]. That potential remains largely prospective, but recent anti-CRISPR engineering in Cas12a systems offers proof of principle that Acrs can be repurposed into programmable switches and spatiotemporal control modules [88, 89]. For Cas13, the priorities are to expand the validated inhibitor repertoire, define ortholog specificity, and determine whether specific inhibitory checkpoints can suppress collateral activity without completely abolishing programmable on-target cleavage [3, 35, 74, 82, 90].

## Harnessing the RNA-targeting versatility: from nuclease-driven destruction to scaffold-mediated regulation

**Figure 4.**
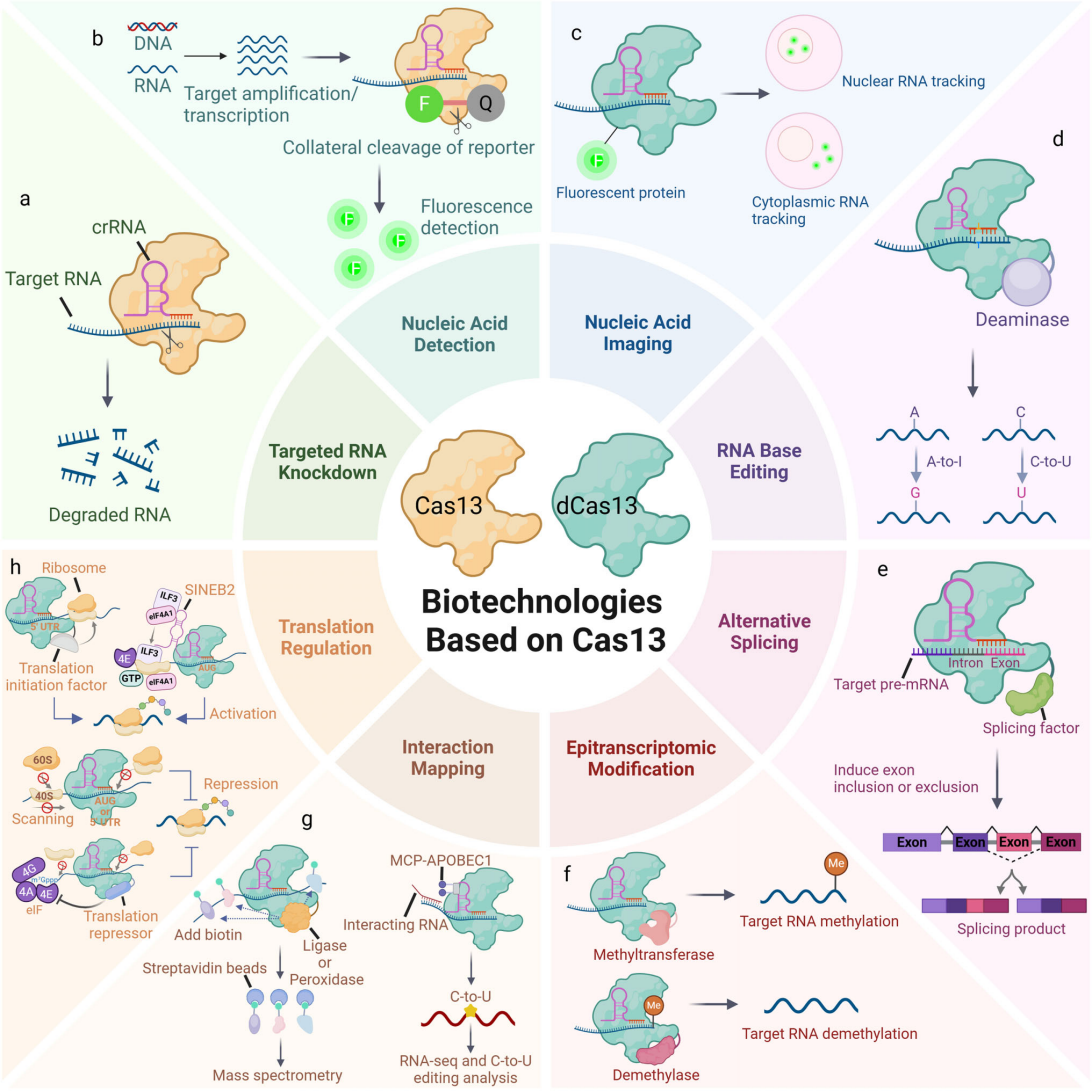
Biotechnological applications of Cas13 and dCas13. (**a**) Programmable RNA knockdown. Targeted transcript degradation is mediated by Cas13-catalyzed cleavage. (**b**) Cas13-based nucleic acid detection. Upon target recognition, the activated Cas13 complex triggers collateral cleavage of fluorophore-quencher labeled ssRNA probes to generate a fluorescent signal. (**c**) RNA imaging and tracking. Fusion of dCas13 with fluorescent proteins enables the spatiotemporal visualization of specific transcripts in live cells. (**d**) Site-directed RNA base editing. dCas13-deaminase fusions facilitate precise nucleotide conversions, including A-to-I and C-to-U, at targeted RNA positions. (**e**) Alternative splicing modulation. Binding of dCas13 to splice sites or recruitment of splicing factors allows for the control of exon inclusion or exclusion. (**f**) Epitranscriptomic editing. Targeted installation or removal of RNA modifications is achieved through dCas13-effector fusions. (**g**) Mapping of RNA-protein and RNA-RNA interactions. In the left panel, proximal protein biotinylation via dCas13-linked ligases or peroxidases enables the identification of RBPs. In the right panel, dCas13-guided C-to-U editing of neighboring RNAs facilitates the discovery of RNA-RNA partners via sequencing analysis. (**h**) Programmable translation regulation. Translation activation (top) is achieved by recruiting ribosomal subunits through dCas13-eIF fusions or the dCasRx-SINEB2 system. Translation inhibition (bottom) is mediated by the physical blockade of ribosome scanning or the recruitment of translational repressors to prevent eIF binding to the m7G cap. eIF, eukaryotic initiation factor; ILF3, interleukin enhancer binding factor 3; SINEB2, short interspersed nuclear element B2. Created in BioRender. Huang, S. (2026) https://BioRender.com/urpibdf

The functional plasticity of CRISPR-Cas13, anchored by its programmable RNA-binding and catalytic versatility, has driven a paradigm shift from conventional RNA interference to sophisticated transcriptomic engineering. Cas13 applications now separate naturally into two primary modalities: catalysis-dependent RNA degradation and catalysis-independent RNA modulation (Fig. 4). The former leverages *cis*- or *trans*-cleavage for targeted knockdown, molecular diagnostics, or counterselection, whereas the latter repurposes Cas13 as a programmable scaffold to recruit RNA-editing enzymes, imaging modules, or regulatory effectors. This progression reflects the field’s maturation from proof-of-concept studies to a robust engineering phase, where maximal effector potency is rigorously balanced against the imperatives of specificity, reversibility, and systemic safety.

### Nuclease-active systems: harnessing the cleavage potential of Cas13

Catalytically active Cas13 has evolved into a versatile RNA knockdown platform across diverse biological systems, spanning from prokaryotes to eukaryotic embryos [21–23, 28, 31, 32, 36, 40, 61, 91]. In 2017, Abudayyeh *et al*. demonstrated that LwaCas13a efficiently knocks down endogenous transcripts in mammalian cells with efficacy comparable to short hairpin RNA (shRNA) but with significantly lower off-target activity, notably extending its utility to nuclear non-coding RNAs [61]. Cas13d subsequently emerged as the dominant mammalian knockdown scaffold due to its compact architecture, high silencing potency, and favorable specificity [22, 65, 92].

While Cas13d exhibits superior knockdown efficiency, its collateral cleavage within eukaryotic systems remains a focal point of academic debate. Translational research increasingly views this *trans*-cleavage activity as a potential driver of cellular homeostasis disruption. Consequently, engineering efforts have pivoted from prioritizing absolute potency toward ensuring stringent *cis* specificity. This pivot is exemplified by the development of ultra-compact orthologs and engineered variants, such as Cas13X/Y, Cas13e, and the miniature Lep/ChiCas13j (424–529 aa), which exploit structural constraints to limit HEPN domain accessibility to non-target RNAs, thereby maintaining high-efficiency knockdown while minimizing global transcriptomic perturbations [31, 32, 36, 40]. Beyond linear messenger RNA (mRNA), Cas13 enables high-fidelity silencing of circular RNAs (circRNAs) by targeting unique back-splicing junctions (BSJs). RfxCas13d-mediated BSJ targeting facilitates >80% circRNA knockdown with minimal impact on cognate linear isoforms, outperforming shRNA in functional screening and oncogenic models [93–95]. Furthermore, codon-optimized variants like Cas13FX extend the platform’s reach to subcellular compartments; for instance, mitochondrial-localized Cas13FX achieves 75%–80% knockdown of mitochondrial transcripts, bypassing the inherent limitations of RNAi within these organelles [64].

Cas13-based molecular diagnostics leverage target-triggered collateral *trans*-cleavage to achieve ultrasensitive detection. The foundational elucidation of Cas13a’s dual, mechanistically distinct RNase activities by East-Seletsky *et al*. established a picomolar (pM) limit of detection (LOD), providing the theoretical framework for subsequent platform engineering [38]. By synergizing Cas13 with isothermal amplification (e.g. RPA), the SHERLOCK platform achieved attomolar (aM) sensitivity and single-base specificity, enabling diverse applications from viral strain differentiation to the detection of cell-free tumor DNA [96–99]. Subsequent iterations, including SHINE (Streamlined Highlighting of Infections to Navigate Epidemics), SHERLOCKv2, CARMEN, and the IMACC microfluidic accelerator, have transitioned the field toward high-throughput multiplexing, one-pot workflows, and robust deployment in clinically relevant environments [26, 98, 100, 101]. This trajectory highlights a shift toward spatiotemporal integration, aiming to reconcile the trade-off between extreme sensitivity and the pragmatic requirements of point-of-care testing (POCT). The current competitive frontier lies in robust signal discrimination within complex biological matrices; overcoming interference in crude clinical samples remains the pivotal hurdle to determining whether Cas13-based technologies can ultimately supersede gold-standard quantitative polymerase chain reaction (PCR).

Phage genome engineering has long been constrained by conventional DNA-targeting CRISPR systems, which often fail to bypass phage anti-defense mechanisms, including DNA modifications, anti-CRISPR proteins, and genome segregation [102]. The RNA-targeting Cas13 system, characterized by PAM independence and broad-spectrum antiphage activity, offers a novel solution. In 2022, Adler *et al*. established a two-step markerless editing workflow via homologous recombination and Cas13a counterselection, enabling 100% efficient genome editing across distinct phages, with edits spanning single-codon substitutions to large multi-gene deletions [103]. Concurrently, Guan *et al*. coupled homologous recombination with Cas13a counterselection using acrVIA1 as a positive selection marker to precisely engineer intractable jumbo phages resistant to DNA-targeting systems [104]. The application of Cas13 has transformed phage engineering from the intractability associated with direct DNA modification into precise selection enabled by RNA degradation. This represents not merely a tool replacement, but a strategic paradigm shift from genome defense to transcriptome intervention.

### Nuclease-inactive systems: dCas13 as a universal RNA-binding platform

Site-specific mutagenesis of the HEPN domains yields catalytically inactive Cas13 (dCas13), which retains programmable RNA-binding capacity while being devoid of endonuclease activity. This transition marks a functional shift from a molecular scissor to a programmable regulatory scaffold, establishing dCas13 as a versatile RNA-targeting modular platform. When fused to diverse functional proteins such as deaminases, epitranscriptomic modifiers, or fluorescent proteins, dCas13 facilitates robust applications in base editing, live-cell RNA imaging, and splicing regulation. This expansion from RNA degradation to precise regulation necessitates sophisticated system-level integration to synergistically optimize both targeting precision and effector catalytic kinetics.

#### Dynamic imaging and interaction mapping: visualizing the RNA life cycle

Fluorescent-protein fusions of dCas13, guided by specific crRNAs, enable the labeling of target RNAs in living cells. Compared to FISH or MS2-MCP systems, dCas13 imaging circumvents the need for chemical fixation, supports long-term tracking (ranging from hours to days), and eliminates the requirement for exogenous RNA tags, thereby minimizing the perturbation of native RNA function. To enhance signal-to-noise ratios, current designs integrate negative-feedback loops or signal-amplification modules to suppress background noise from unbound effectors, facilitating the dynamic visualization of mRNA transport and nuclear lncRNA localization [61]. The platform’s versatility is further expanded by orthologous Cas13 variants, aptamer-modified guides, and “LiveFISH” strategies, which enable multicolor RNA imaging and integrated RNA–DNA spatiotemporal visualization [105–108]. More sophisticated architectures, such as the dCas13a-SunTag-bimolecular fluorescence complementation (BiFC) system, synergize SunTag-mediated amplification with BiFC to achieve exceptional sensitivity, allowing the real-time observation of endogenous cytoplasmic and nuclear RNA dynamics, including discrete splicing events [109]. Ultimately, the paradigm shift offered by dCas13 lies in its capacity to observe native RNA behavior in real-time without the need for irreversible transcriptomic rewriting.

The programmable RNA-binding precision of dCas13 has been repurposed for spatiotemporal interactome profiling through proximity labeling and capture-based proteomics. By fusing dCas13 with enzymatic modules such as APEX2, proximity biotinylation can be directed to the native RNA microenvironment upon H_2_O_2_/biotin-phenol activation, identifying RNA-associated proteins without exogenous tagging [110]. To bolster sensitivity for low-abundance transcripts, the addition of a double-stranded RNA-binding domain from PKR has been shown to stabilize the dCas13–guide RNA (gRNA)–target complex and enhance binding affinity [110]. More specialized platforms, such as CRUIS (leveraging the PUP-IT system) and CARPID (utilizing the BASU ligase), facilitate the covalent labeling of adjacent RBPs, enabling high-fidelity capture of weak or transient interactions across diverse lncRNA lengths and subcellular compartments [111, 112]. Alternatively, CBRPP (CRISPR-based RNA proximity proteomics) employs UV-crosslinking and HA-mediated enrichment to profile native RNA–protein complexes [113]. Beyond RNA–protein interactions, the scope of Cas13-based mapping has expanded to RNA–RNA contacts. For instance, the sarID platform utilizes a dCas13-MCP-APOBEC1 architecture to record proximity-based RNA–RNA contacts via C-to-U editing, offering a crosslinking-free, *in vivo* alternative to traditional RAP-MS or HyPro-seq [114].

Despite these advancements, dCas13-based imaging remains constrained by suboptimal gRNA design tools and the propensity of RNA secondary structures to mask target sites. A more profound concern involves the potential of the bulky dCas13 complex (steric hindrance) to alter the native folding, subcellular localization, and endogenous RBP interactome of the target RNA. Additionally, sporadic non-specific binding can lead to elevated background fluorescence, complicating data interpretation. Future developments will likely prioritize predictive gRNA design algorithms that account for RNA accessibility, optimized labeling strategies to minimize biophysical perturbations, and signal amplification via single gRNAs to enhance detection sensitivity.

#### Precise epitranscriptomic and sequence editing: the “hit-and-run” paradigm

Cas13-based RNA base editing facilitates site-specific nucleotide conversion without inducing permanent genomic alterations by coupling programmable dCas13 with cytidine or adenosine deaminases. The pioneering REPAIR (RNA Editing for Programmable A-to-I Replacement) platform, which fused dCas13b to the catalytic domain of ADAR2 (ADAR2_DD_), enabled programmable A-to-I transitions, where Inosine (I) is functionally interpreted as guanosine (G) by the translational machinery [63]. To broaden the targeting scope, RESCUE (RNA Editing for Specific C-to-U Exchange) expanded the catalytic repertoire of ADAR2_DD_ to mediate both A-to-I and C-to-U editing; however, inherent off-target deamination necessitated further refinement, yielding the higher-fidelity RESCUE-S variant [115]. Alternatively, the CURE (C-to-U RNA Editor) system, utilizing an engineered APOBEC3A domain fused to dPspCas13b or dCasRx, bypasses A-to-I off-target liabilities and provides distinct motif preferences across nuclear and cytoplasmic compartments [116]. To overcome performance plateaus, the eRESCUE system enhances editing efficiencies, though this increased potency correlates with elevated off-target activity [117].

Translational progress has been significantly constrained by cargo size, as numerous Cas13-editor constructs exceed the packaging threshold of single AAV vectors (∼4.7 kb). This challenge has spurred the development of compact editors, including mxABE/mxCBE, REPAIR.t/RESCUE.t, and systems driven by ChiCas13j, which fit within AAV limits [31, 32, 40]. Concurrently, spatiotemporal precision has been achieved using light-inducible platforms (paCas13 and padCas13), enabling blue-light-activated, reversible RNA editing both *in vitro* and *in vivo* [118].

The primary clinical appeal of RNA editing lies in its reversibility, which circumvents permanent genomic alterations. However, this transience necessitates persistent administration to maintain therapeutic levels, potentially triggering host immunogenicity. Consequently, resolving the translational bottleneck requires not only miniaturization but also the integration of conditional activation, enhanced target selectivity, and sustained efficacy within a single design framework.

Post-transcriptional regulation is profoundly governed by dynamic RNA modifications that dictate splicing, translation efficiency, and transcript stability. Among these, N^6^-methyladenosine (m^6^A) is the most prevalent mRNA modification, regulating diverse physiological and pathological processes [119]. To decode this landscape, dCas13 platforms have been engineered to site-specifically interrogate and manipulate these marks. By fusing dCas13 with dedicated modification readers, writers, or erasers, researchers can precisely orchestrate m^6^A deposition, removal, and functional interpretation on native transcripts [120–127]. Beyond m^6^A, the targeting repertoire of dCas13 has rapidly expanded to other critical modifications. For instance, the RCMS system utilizes nuclear dCasRx fusions with NSUN2/NSUN6 or TET2 to mediate m^5^C methylation or demethylation on mRNAs and tRNAs [128]. Similarly, chemically induced proximity systems enable reversible m^1^A editing via ALKBH3 or TRMT61A recruitment, offering precise temporal control through ABA-inducible or light-gated mechanisms [129]. Furthermore, targeted N^4^-acetylcytidine (ac^4^C) installation is achievable via dCas13-eNAT10 fusions, which have been successfully adapted for *in vivo* dual-AAV delivery in murine models [130]. By dictating the localized epitranscriptomic state, these tools empower researchers to dissect the complex regulatory layers bridging genotype and cellular phenotype. Although clinical translation remains nascent, these technologies fundamentally elevate Cas13 from a simple targeting tool to a comprehensive platform for advanced RNA biology.

#### Splicing and translation modulation: post-transcriptional fine-tuning

Alternative splicing, governed by dynamic interactions between *cis*-acting elements and *trans*-acting factors, is a primary engine of proteomic diversity [131]. By programmably binding key splicing elements, dCas13 can sterically interfere with spliceosome assembly or recruit splicing regulators, providing a versatile platform for splicing modulation. For instance, dCasRx can sterically block spliceosome assembly at splice acceptor sites, whereas CASFx-like fusions recruit defined regulatory domains to drive exon inclusion or exclusion [22, 132]. These approaches exhibit substantial translational utility, exemplified by the therapeutic correction of *SMN2* exon 7 in spinal muscular atrophy models, and enable precise control over endogenous protein expression, such as tuning serine/arginine-rich protein levels via poison exon modulation [132, 133].

Beyond *cis*-splicing regulation, dCas13 has been repurposed to orchestrate mRNA *trans*-splicing. The CRAFT (CRISPR Assisted RNA Fragment Trans-splicing) platform synergizes dCas13 with a recombinant *trans*-splicing RNA (rcRNA) carrying corrective fragments to achieve spliceosome-mediated transcript rewriting [134]. Pushing this paradigm further, the dual-CRISPR RESPLICE system utilizes a dCasRx-directed *trans*-splicing module for cargo-target co-localization, coupled with a Cas7-11-directed *cis*-splicing interfering module that suppresses endogenous splicing by cleaving the target pre-mRNA, thereby markedly enhancing *trans*-splicing efficiency [135]. Although these multimeric systems remain technically demanding, they underscore a profound conceptual shift: Cas13 technologies have evolved beyond simple nucleotide degradation or point editing to fundamentally reshape transcript architecture.

The programmable RNA-binding capacity of dCas13 has been extensively repurposed to modulate translation initiation and recoding. Translational activation (CRISPR-RNAa) is achieved by fusing dCasRx to translation initiation factors, which recruits ribosomal subunits to the 5′ UTR to enhance protein output in a position-dependent manner [136]. A parallel strategy, the SINEB2-dCasRx system, mimics antisense lncRNA mechanisms to boost translation without altering mRNA abundance [137, 138]. This approach has demonstrated therapeutic potential *in vivo*, where AAV-mediated delivery activated tumor suppressors such as P53 and PTEN, leading to significant anti-proliferative effects [138]. Conversely, dCas13 facilitates translational repression through steric interference. In prokaryotes, dCas13 targeting the Shine–Dalgarno sequence effectively blocks ribosome assembly, whereas in eukaryotes, the CRISPRδ platform inhibits translation by physically obstructing ribosome scanning [139, 140]. Notably, CRISPRδ can suppress cap-dependent, IRES-dependent, and repeat-associated non-AUG translation, providing a targeted strategy to mitigate pathogenic protein synthesis in neurodegenerative diseases like *C9orf72*-associated ALS/FTD [140]. Beyond initiation, dCas13 has been engineered to modulate stop codon readthrough. By binding downstream of a termination site, dCas13 stalls advancing ribosomes, increasing the probability of readthrough at premature termination codons (PTCs) [141]. This “ribosome-stalling” mechanism offers a promising framework for correcting PTC-associated pathologies, including thalassemia and hereditary spherocytosis [142].

These applications exemplify the functional evolution of Cas13 from a binary “switch” to a tunable “rheostat.” For genetic disorders characterized by haploinsufficiency, dCas13-mediated translational upregulation provides a homeostatic alternative to conventional gene augmentation. By modulating endogenous transcripts, this approach operates within the constraints of native regulatory networks, preserving physiological feedback loops and mitigating the risks of ectopic overexpression.

The bifurcation of Cas13 into nuclease-dependent degradation and nuclease-inactive modulation defines the dual trajectory of programmable RNA technologies. As the field matures from tool expansion toward mechanistic refinement, a fundamental challenge persists: the structural decoupling of ancestral viral defense mechanisms, specifically collateral activity, from the requirements of precision bioengineering. The future of Cas13-based medicine likely hinges on the development of sophisticated conditional systems, where activity is gated by light, chemical inducers, or cell-specific contexts, shifting the paradigm from static tools toward dynamic, RNA-regulating logic gates.

## Clinical translation of Cas13: from diagnostics to therapeutics

**Figure 5.**
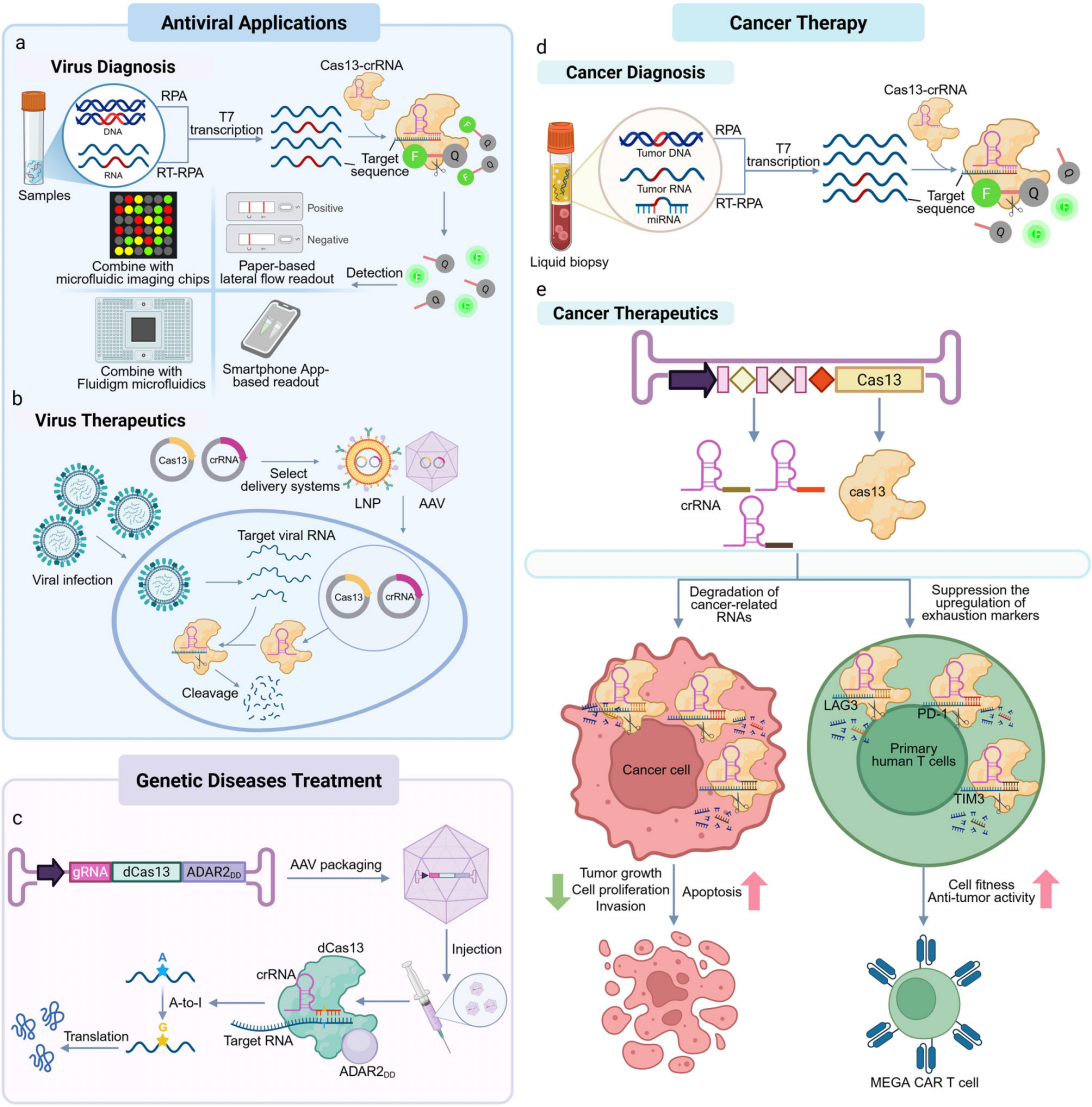
Cas13-based disease diagnosis and therapy. (**a**) Viral diagnostics. Nucleic acids are amplified via RPA or RT-RPA and transcribed by T7 polymerase. Recognition of the target RNA activates Cas13 collateral cleavage of fluorophore-quencher probes, enabling detection via diverse platforms such as lateral flow assays, mobile readers, and microfluidic systems. (**b**) Antiviral applications. Following the delivery of specific crRNAs, the Cas13 system degrades viral RNA to inhibit infection and replication. (**c**) Genetic disease therapy. AAV-mediated delivery of dCas13-ADAR fusions allows for the reversible correction of mutations in target transcripts through A-to-I or C-to-U base editing, thereby restoring functional protein expression. (**d**) Cancer diagnostics. Tumor markers, including circulating DNA, RNA, and microRNAs (miRNAs), are isolated from liquid biopsies and amplified. Target recognition by the Cas13 complex triggers reporter cleavage to generate detectable fluorescence signals for cancer identification. (**e**) Cancer therapeutics. Cas13 can be programmed to target oncogenic RNAs, leading to the inhibition of tumor growth and the induction of apoptosis. Additionally, Cas13-mediated knockdown of CAR T cell exhaustion markers, such as LAG3, PD-1, and TIM3, improves T cell fitness and anti-tumor activity through the generation of multiplexed effector guide arrays (MEGA) CAR T cells. Created in BioRender. Huang, S. (2026) https://BioRender.com/urpibdf

The clinical translation of CRISPR-Cas13 harnesses its dual functional modalities: target-triggered *trans*-cleavage facilitates ultrasensitive diagnostic signal amplification, while *cis*-cleavage and dCas13-mediated transcript modulation offer diverse therapeutic avenues (Fig. 5). In both contexts, transitioning from proof-of-concept validation to clinical robustness remains the primary bottleneck. The central translational challenge has shifted from demonstrating fundamental efficacy in experimental models to ensuring the predictability and safety of Cas13 performance within complex clinical matrices, physiologically diverse tissues, and across longitudinal dosing regimens.

### Next-generation diagnostics and biomarker discovery: pushing the limits of sensitivity and integration

The diagnostic paradigm of Cas13 relies on converting a target-recognition event into an exponential signal cascade via target-triggered *trans*-cleavage. This mechanism has established a new frontier for infectious disease monitoring and cancer liquid biopsies, addressing the inherent limitations of traditional PCR-based workflows.

#### Evolution from amplification-coupled to amplification-free platforms

Cas13-based diagnostics have evolved from initial amplification-coupled assays to high-throughput multiplexing and direct digital quantification. Standard platforms, such as SHERLOCK, integrate Cas13 with isothermal amplification (e.g. reverse transcription RPA, RT-RPA) to detect pathogens such as SARS-CoV-2, Ebola, and dengue with aM sensitivity, often streamlined by extraction-free lysis (Heating Unextracted Diagnostic Samples to Obliterate Nucleases, HUDSON) and one-pot formats like SHINE and CESSAT (Chemical additive-Enhanced Single-Step Accurate CRISPR/Cas13 Testing system) to facilitate rapid POCT [96, 98, 143–152].

This high-fidelity sensing extends to oncology, where SHERLOCK can distinguish SNVs, including *EGFR* L858R or *BRAF* V600E, in liquid biopsies with mutant allele frequencies as low as 0.1% [96, 100]. To expand diagnostic breadth, SHERLOCKv2 incorporates Csm6-mediated amplification and multi-ortholog detection, while microfluidic platforms like CARMEN enable massive parallelization to profile thousands of viral targets or drug-resistance mutations on a single chip [100, 153].

Concurrently, the field is transitioning toward amplification-free and digital detection to minimize bias and simplify clinical implementation. Platforms such as FIND-IT deliver results in under 30 min, while digital partitioning systems like STAMP and electrochemical sensors like COMET or SCOPE achieve sub-attomolar quantification of scarce biomarkers, including miRNA and extracellular-vesicle mRNA, directly from complex clinical matrices [154–161]. While the trajectory focuses on digitizing the assay to bypass pre-amplification, achieving clinically relevant LOD remains biophysically constrained by the diffusion limits of Cas13 and target RNAs in crude samples.

#### Cas13 as a discovery engine for functional biomarkers

Beyond detection, Cas13 functions as a powerful discovery engine by bridging the gap between identification and clinical validation. Platforms such as CLISA leverage Cas13-mediated reporter amplification to substantially improve the readouts of low-abundance proteins [162]. Similarly, transcriptome-wide Cas13d screening has facilitated the discovery of functional biomarkers and therapeutic targets, particularly in the study of lncRNAs in cancer [163, 164]. In these contexts, transcript-level perturbations reveal dependencies that are often poorly captured by DNA-centric approaches

This integrated capacity is particularly valuable for liquid biopsies, where the pivotal question is not merely whether a biomarker is present, but whether it reflects a biologically actionable RNA state. While rare mutant transcripts and multiplexed miRNA signatures are often masked by high background, Cas13 provides a modular combination of design flexibility and signal amplification. Ultimately, in a clinical landscape where biomarker panels evolve rapidly, this adaptability may prove as critical as absolute sensitivity for the implementation of next-generation diagnostic workflows.

### Therapeutic interventions: balancing transcriptomic precision and systemic safety

Conceptually, Cas13 offers a distinct advantage over permanent genome editing: the reversible, dosage-tunable manipulation of pathogenic RNA. This principle is particularly compelling across three major domains: antiviral therapy, gene expression-related disorders, and oncology. However, clinical success in these areas necessitates the simultaneous resolution of delivery efficiency, guide performance, collateral activity control, and immunological tolerance.

#### Antiviral strategies: the race against viral evolution

Current countermeasures against RNA viruses, primarily vaccines and small-molecule antivirals, are frequently undermined by rapid viral evolution and protracted development cycles. CRISPR-Cas13 offers a programmable alternative, leveraging precise crRNA design to target diverse pathogens across viral families. CARVER established a dual framework for Cas13-mediated viral RNA inhibition and SHERLOCK-based viral readout, whereas PAC-MAN demonstrated programmable targeting of SARS-CoV-2 and live influenza A virus in cellular models [165–167]. Bioinformatic modeling further suggests that a minimal panel of conserved crRNAs could enable “pan-coronavirus” strategies, providing broad-spectrum coverage that traditional vaccines often lack [167]. Subsequent Cas13 studies further supported broad-spectrum inhibition of SARS-CoV-2 variants and endemic coronaviruses *in vitro* and in airway epithelial culture models [168].

Cas13 antiviral activity has been supported across cellular, *ex vivo*, and various animal models of infection. For instance, lipid nanoparticles (LNPs)-encapsulated Cas13 mRNA significantly reduces viral titers and ensures survival in dengue virus (DENV)-infected mice and influenza-infected hamsters; virus-like particles can deliver PspCas13b-crRNA RNPs into primary human cells at nanomolar concentrations to inhibit DENV; and AAV-mediated delivery against enterovirus A71 (EV-A71) has achieved 100% survival in lethal challenges [169–177]. Beyond human therapeutics, the technology extends to vector control via systems like REAPER, which utilizes Cas13 collateral activity to eliminate infected mosquitoes and curtail transmission [178, 179].

Cas13 is particularly advantageous in outbreak scenarios or rapidly evolving viral landscapes, as guide panels can be redesigned significantly faster than traditional small-molecule discovery pipelines. Yet, this speed advantage only translates to clinical utility if coupled with scalable delivery and rigorous resistance management. Consequently, multiplexed guides, conserved-region targeting, and optimized dosing regimens must be treated as essential design foundations rather than optional refinements. Ultimately, clinical antiviral efficacy hinges on the synergy between programmable guide design and delivery systems capable of reaching target cells before viral replication outruns therapeutic intervention. This kinetic competition, rather than mere enzymatic efficiency, remains the primary hurdle for Cas13-based antivirals. Given the exponential nature of viral propagation, therapeutic constructs must achieve rapid, comprehensive biodistribution before titers exceed the threshold for effective cellular intervention. Furthermore, while targeting conserved sequences minimizes escape, the intense selective pressure of Cas13 cleavage may catalyze unpredictable evolutionary shifts, necessitating multiplexed guide strategies to ensure long-term therapeutic durability.

#### Therapeutic strategies for gene expression-related disorders

For genetic and transcriptopathy-focused applications, Cas13 functions through three primary modalities: RNA base editing, allele-selective transcript depletion, and splicing correction. Compact systems like mxABE, Chi-RESCUE-S, and hfCas13x.1 have effectively circumvented AAV packaging constraints, enabling successful *in vivo* correction in models of Duchenne muscular dystrophy, hearing loss, and inherited retinal diseases [32, 40, 180–183]. Photoactivatable editors like PA-rABE further enhance precision, allowing for light-mediated repair of hemophilia B mutations with minimal bystander effects [184]. Beyond editing, nuclease-active Cas13 variants provide a mechanism to silence pathogenic RNAs or discriminate SNVs. This allele-selective depletion has rescued phenotypes in Angelman syndrome by targeting *UBE3A*-*ATS* and familial cardiomyopathies by selectively degrading mutant *MYH7* or *TNNT2* transcripts [185–187]. Such precision extends to neurodegenerative repeat-expansion diseases (e.g. *C9orf72*-associated ALS/FTD) and ocular conditions, where Cas13-mediated knockdown of *VEGFA* or *AQP1* reduces pathological neovascularization and intraocular pressure [188–191]. Besides, RfxCas13d-mediated transient activation of Wnt/β-catenin signaling via simultaneous degradation of *Axin1/2* mRNAs promotes alveolar regeneration and suppresses bleomycin-induced fibrosis [192]. Splicing correction remains a particularly attractive niche, as it directly exploits the RNA-centric nature of Cas13 to shift exon inclusion or skip pathogenic variants, as demonstrated in models of spinal muscular atrophy, frontotemporal dementia, and Hutchinson–Gilford progeria syndrome [22, 132, 193].

Collectively, these advancements underscore that Cas13 is best suited for RNA-dominant pathologies where transient or titratable modulation is preferable to irreversible DNA editing. The translational frontier remains centered on the synergy between precise transcript modulation and scalable, tissue-specific delivery.

#### Precision oncology remediation

In oncology, Cas13-based platforms leverage programmable transcriptomic intervention to bridge the gap between high-precision oncogene silencing and targeted cytotoxic activation, a versatile repertoire that extends from the selective depletion of mutant alleles and oncogenic transcripts to the reshaping of tumor-supporting RNA programs and the transient engineering of immune-cell states [68, 194–198]. By targeting an array of drivers, including mutant *KRAS, EML4*-*ALK* fusions, and oncogenic lncRNAs, Cas13 can effectively suppress proliferation and induce apoptosis across various malignancies [195, 198]. Recent engineering refinements have further sharpened this precision; for instance, the introduction of synthetic mismatches allows crRNAs to discriminate SNVs with unprecedented allele selectivity, enabling the silencing of oncogenic variants like *BRAF* V600E while sparing wild-type transcripts [60]. Beyond intrinsic tumor targeting, the MEGA platform demonstrates a distinct translational route in cell therapy, utilizing reversible multiplex knockdown to tune CAR-T fitness and exhaustion without the risks of permanent genomic editing [27].

The most compelling aspect of Cas13 in oncology, however, lies in its catalytic duality. While conventional modalities emphasize *cis*-cleavage for knockdown, emerging paradigms exploit target-triggered collateral cleavage to elicit localized transcriptomic collapse within malignant cells. This “bystander killing” can be gated by tumor-specific triggers, such as *EGFRvIII* expression or high intratumoral ATP levels sensed through aptamer-based non-canonical activation [68, 199]. To maximize *in vivo* efficacy, Cas13 platforms are increasingly integrated with advanced delivery vehicles and combinatorial regimens. Such “smart” delivery systems, including hierarchical self-uncloaking nanostructures that release therapeutics upon viral or oncogenic RNA sensing, allow Cas13 to function as a conditional biological logic gate, transforming tumor-specific markers into lethal triggers [200]. This spatial precision is further augmented by targeted delivery innovations. For example, tumor-targeting liposomes delivering Cas13a to bladder cancer cells enabled the multiplexed knockdown of critical survival factors, significantly reducing *in vivo* tumor burden [194]. To further enhance potency, the AAV-compatible CCRS platform integrates CasRx with antisense ribozymes via synthetic fusion gRNAs, outperforming standalone CRISPR or ribozyme approaches in suppressing metastasis [201]. Moreover, Cas13 is increasingly incorporated into combinatorial regimens. For instance, the intravesical co-delivery of Cas13a-targeting PD-L1 with fenbendazole nanoparticles has been shown to synergistically remodel of the immune microenvironment and promote apoptosis, yielding tumor inhibition and markedly extending survival in orthotopic bladder cancer models [202].

Despite technical progress, transitioning Cas13 from proof-of-concept to clinical application remains limited by biological and immunological barriers. Notably, pre-existing immunity to RfxCas13d, characterized by IgG antibodies and inflammatory cytokine responses (e.g. IFN-γ, TNF-α, and IL-17), represents a substantial hurdle for repeated dosing [203]. The translational value of Cas13 likely lies in specific scenarios where transient, adjustable, or multiplexed RNA modulation offers clear advantages over permanent DNA editing, or where it can be combined with current chemotherapy and immunotherapy to modify the tumor microenvironment. Successful application of Cas13 in oncology will depend on balancing its functional flexibility with rigorous immunological and delivery control.

## Challenges and future perspectives: a critical outlook on clinical translation

As a programmable RNA-targeting platform, CRISPR-Cas13 offers a transformative modality for transient and reversible therapeutic intervention. However, its broad clinical adoption is fundamentally contingent upon resolving the tripartite challenges of delivery efficiency, targeting precision, and immunological compatibility.

### The delivery-activity nexus: navigating physical and biological constraints

The transition of Cas13 from *in vitro* validation to *in vivo* efficacy is primarily bottlenecked by the inherent limitations of current delivery vehicles. AAV vectors are familiar and efficient but impose stringent payload limits that force difficult trade-offs between enzyme size, effector fusion complexity, and regulatory elements [32, 40]. The current research focus on ultra-compact variants often necessitates a compromise, potentially reducing catalytic activity or altering specificity profiles, representing a critical juncture in protein engineering. Simultaneously, although non-viral platforms such as LNPs and polymeric nanoparticles (e.g. P76, a lung-targeted polymer formulation, for pulmonary nebulization) circumvent capacity limits, their lack of intrinsic tissue tropism requires the development of hierarchical, “smart” delivery systems [202, 204, 205]. This deficit in targeting necessitates the conjugation of specific ligands or the engineering of stimuli-responsive formulations, which complicates manufacturing and elevates concerns regarding off-target exposure and organ-specific toxicity. While tailored solutions, such as fluorinated chitosan for intravesical delivery or stimuli-responsive RNA nanococoons (RNCOs-D), illustrate how delivery can be adapted to specific anatomical niches, these complex architectures introduce formidable hurdles regarding manufacturing scalability, batch-to-batch consistency, and stringent regulatory approval [200].

Consequently, the synergy between delivery vehicles and enzyme architecture will define the next phase of Cas13 development. The primary engineering priority has shifted from merely achieving *in vivo* delivery to identifying the optimal constellation of ortholog, effector, and delivery method suited to specific target tissues and therapeutic windows. This integrated approach represents a more sophisticated design problem than simple proof-of-principle editing, yet it is the essential evolution required for Cas13 to move beyond laboratory settings toward predictable, clinically relevant outcomes.

### Deciphering the collateral conundrum: from toxicity to tunability

The collateral *trans*-cleavage activity that powers Cas13 diagnostics remains its primary therapeutic liability. Emerging evidence indicates that this effect is not a binary “on/off” phenomenon but is highly dose-dependent: while high-level expression of variants like RfxCas13d can trigger broad transcriptomic degradation, low-dose delivery often achieves efficient on-target knockdown with negligible bystander effects [206]. This realization shifts the safety paradigm from a singular focus on high-fidelity variants, which often sacrifice potency for specificity, toward an integrated strategy that precisely governs the magnitude and duration of enzyme expression.

Beyond protein redesign, safety can be bolstered through optimized guide design (e.g. Cas13Design, a transcriptome-wide guide design tool), chemically modified gRNAs, and the recruitment of tissue-specific promoters or anti-CRISPR proteins to modulate *in vivo* activity [53, 58, 206, 207]. Structurally, identifying and modifying domains involved in non-specific RNA engagement offers a promising path to mitigating collateral risks, analogous to the engineering of Cas9-family high-fidelity nucleases [208]. However, because off-target profiles vary significantly across subtypes and cellular environments, comprehensive transcriptome-wide profiling in relevant preclinical models remains a non-negotiable prerequisite for clinical translation. Ultimately, the success of Cas13 therapeutics hinges on a coordinated framework that preserves robust *cis*-cleavage while successfully suppressing deleterious *trans*-activity.

### The immunological and regulatory frontier

As bacterial-derived proteins, Cas13 effectors face significant hurdles regarding both innate and adaptive immune recognition. Pre-existing immunity to common orthologs, such as RfxCas13d, has already been documented in human populations, raising concerns for inflammatory toxicity and diminished efficacy during repeated dosing [203]. Beyond classical immunogenicity, non-canonical behaviors such as RNA target-independent non-canonical activation further complicate the safety profile by potentially triggering non-specific RNA degradation [199]. These risks necessitate a profound mechanistic understanding to prevent unintended transcriptomic damage. This landscape reinforces the value of transient expression as a core design principle. Strategies such as mRNA delivery, timed expression windows, and reversible anti-CRISPR control allow for tightly bounded activity, minimizing the risks associated with the long-term persistence of foreign proteins in the host.

### The Cas13 versus Cas7-11 dialectic: selecting the right tool for the task

Beyond the Cas13 family, the Type III-E effector Cas7-11 has emerged as a significant alternative for programmable RNA targeting [209]. Composed of seven functional domains, Cas7-11 utilizes its Cas7.2 and Cas7.3 subunits to catalyze precise target cleavage without detectable collateral activity under tested conditions [210]. This lack of bystander effects, rooted in a strict dependence on specific base-pairing positions, makes Cas7-11 an intrinsically attractive candidate for applications where absolute precision is paramount. However, recent comparative studies in zebrafish embryos indicate that Cas7-11 currently lacks the potency of its predecessors, achieving knockdown efficiencies significantly lower than those of RfxCas13d [207]. Despite the precision of Cas7-11, Cas13 remains the more mature and widely deployed platform due to its superior targeting flexibility, higher potency, and established engineering infrastructure. Its functional versatility enables a spectrum of applications ranging from ultra-sensitive diagnostics, such as SHERLOCK, to advanced RNA editing and live-cell imaging. Furthermore, the naturally low molecular weight of subtypes like Cas13d provides a distinct advantage for viral vector packaging. Ultimately, the choice between these platforms is application-specific rather than a matter of universal superiority. While Cas7-11 is preferable for scenarios requiring maximum cleavage precision, Cas13 remains the cornerstone of RNA research and translation due to its technical maturity and versatility. The future of RNA engineering will likely involve a complementary toolkit, where the strengths of both systems are leveraged to meet specific research and clinical needs.

### Conclusion and future trajectories

Cas13 has evolved from a prokaryotic immune effector into a multifaceted RNA engineering platform, with its true strength lying in its programmability across diverse biological contexts. The field is now shifting from rapid tool expansion toward a more disciplined, integration-focused phase. Future progress will likely be defined by three key technological pillars: (i) artificial intelligence (AI)-guided design: utilizing deep learning to predict not only gRNA efficiency but also subcellular RNA-binding dynamics and collateral cleavage risks; (ii) multimodal combination therapies: synergizing Cas13-mediated knockdown with existing small-molecule or immunotherapy regimens to overcome drug resistance and enhance therapeutic depth; and (iii) transient delivery paradigms: prioritizing non-viral, transient expression systems to minimize the temporal footprint of Cas13 and reduce long-term immunogenicity.

Achieving clinical translation will require moving beyond isolated improvements toward a systems-level engineering approach. This involves coupling a deeper mechanistic understanding of Cas13 activation with delivery modalities that align exposure with specific tissues and timeframes. While significant obstacles in delivery and biosafety persist, the convergence of protein engineering, computational biology, and material science is progressively moving Cas13 closer to a clinically tractable RNA-targeting platform.

Ultimately, the success of the next stage will be measured not by the proliferation of new orthologs, but by the ability to integrate subtype-aware design with controllable catalytic states and realistic delivery frameworks. Even as individual tools evolve, the core logic established by the Cas13 platform of treating RNA as a dynamic and programmable substrate for reversible manipulation is poised to outlast any single variant and will continue to shape the future of RNA therapeutics and diagnostics.

## Data Availability

No new data were generated or analyzed in support of this research.
